# Anxiety, symptoms of temporomandibular disorders (TMD), and awake bruxism in children: an observational study

**DOI:** 10.1007/s40368-026-01211-0

**Published:** 2026-05-13

**Authors:** L. A. M. Torres, M. V. Heimer, F. J. C. de Lima, B. L. Donato, J. F. O. L. Batista, G. P. Falcão, J. V. Silva, L. L. O. Barbosa, V. E. dos Santos-Junior

**Affiliations:** 1 Faculty of Dentistry, Federal University of Alagoas, Maceió, Brazil; 2 Faculty of Dentistry, Universidade de Pernambuco, Recife, Brazil

**Keywords:** Anxiety, Bruxism, Temporomandibular disorders, Awake bruxism, Childhood

## Abstract

**Objectives:**

The aim of this study was to evaluate the association among anxiety, symptoms of temporomandibular disorders, and awake bruxism in children.

**Methods:**

A cross-sectional study was carried out with 274 children aged between 7 and 12 years. Data were collected through structured questionnaires, applied by interview and clinical assessment. Statistical analysis involved descriptive and inferential analysis using the Chi-square test and Fisher’s exact test. A binary logistic regression model was applied to identify independent predictors of anxiety.

**Results:**

Age ranged from 7 to 12 years and was equally distributed between the sexes. 23.3% of the sample presented anxiety; 4.3% presented TMD symptoms; 18.2% presented bruxism. Anxiety was associated with sex (*p* = 0.005), TMD symptoms (*p* = 0.008), and tooth wear (*p* = 0.005). In the logistic regression model, tooth wear, TMD symptoms, and male sex remained independent predictors of anxiety, whereas awake bruxism and age were not. The model explained a limited proportion of the variance in anxiety.

**Conclusion:**

Anxiety was observed in nearly one-quarter of the study sample and was associated with sex, TMD symptoms, and tooth wear. Awake bruxism, however, was not an independent predictor of anxiety. Given the cross-sectional design and the limited variance, these findings should be interpreted as exploratory and do not support causal inferences.

## Introduction

According to the World Health Organization, childhood comprises the first decade of life, with school-age children are defined as those between 6 and 12 years of age (WHO, 2020). This period is marked by intense physical, emotional, and cognitive development, during which emotional regulation mechanisms are still maturing (Benton et al. 2021). Consequently, psychosocial factors, particularly anxiety, may exert a significant influence on children’s overall health, behaviors, and adaptations to daily demands (Viswanathan et al. 2022; Villanueva et al. 2024).

In a biopsychosocial context, anxiety may relate to children’s emotional regulation and health-related behaviors (Rask et al. 2024). Anxiety is among the most prevalent emotional conditions in childhood and is characterized by excessive fear, irritability, restlessness, sleep disturbances, and difficulty concentrating (Vallance and Fernandez 2016). When persistent, anxiety can impair children’s social interaction, academic performance, and cognitive development, and may predispose individuals to psychiatric disorders in adulthood (Vallance and Fernandez 2016). Additionally, anxiety has been associated with various multifactorial conditions, including temporomandibular disorders (TMD), which may both influence and be influenced by emotional dysregulation (Manfredini et al. 2025).

TMD comprise a group of musculoskeletal conditions involving the temporomandibular joint, masticatory muscles, and associated structures (Manfredini et al. 2025). According to INfORM/IADR 2025, TMD are understood within a multifactorial framework involving interactions among biological, mechanical, and psychosocial factors. A comprehensive diagnosis of TMD requires consideration of the patient’s medical history, a detailed clinical examination by a qualified professional, and the individual’s subjective perception of symptoms (Manfredini et al. 2025). It is also essential to assess the presence of co-existing conditions that may influence symptom presentation or prognosis, such as anxiety and bruxism (Verhoeff et al. 2025).

In turn, bruxism is defined as repetitive masticatory muscle activity, which may manifest as clenching, grinding, or mandibular bracing. This activity can occur during both nocturnal sleep and wakefulness; therefore, it is classified as sleep or awake bruxism. It is important to emphasize that awake bruxism and sleep bruxism are distinct behaviors with different underlying mechanisms and psychosocial associations (Bracci et al. 2023; Verhoeff et al. 2025).

According to the International Consensus of Bruxism, although this behavior is centrally regulated, it cannot be classified as a disorder or disease per se, as this classification depends on the presence of additional associated factors. Assessment can be performed through clinical evaluation, patient self-report, or complementary examinations such as polysomnography, which is regarded as the gold standard. In children, bruxism may be related to emotional dysregulation, sleep fragmentation, environmental exposures, such as passive smoking, and still-maturing neuromuscular control (Manfredini et al. 2025; Abrahams et al., 2023, Verhoeff et al. 2025). Moreover, bruxism has been described as being associated with anxiety and TMD and it may contribute to harmful clinical consequences such as increased muscle activity and tooth wear (Verhoeff et al. 2025). When referring specifically to awake bruxism (AB), the behavior is more often characterized by clenching rather than grinding, which is why it is commonly associated with painful muscular TMD (Bracci et al. 2023). Prevalence estimates vary widely, from 6 to 24% in young individuals (Zielinski et al., 2024).

Bruxism in childhood may represent one of the earliest entry points into the broader medicalization of dental practice, aligning with the ongoing shift from technically driven approaches toward comprehensive, evidence-based evaluation. In this context, bruxism has been proposed as a clinical gateway to dentistry, as its signs and symptoms, such as tooth wear, muscle fatigue, or morning jaw discomfort, are often among the first reasons prompting children and their caregivers to seek dental evaluation (Manfredini 2024). However, this perspective remains largely conceptual, and its applicability with pediatric populations requires further empirical support.

Damage to dental structures may appear in several forms, including marked incisal and occlusal wear due to the lower mineralization of primary teeth. Other clinical signs include tooth fractures, thickening of the periodontal ligament, linea alba, dental impressions on the tongue, and traumatic soft tissue injuries. Children with bruxism may also present discomfort or pain in the temporomandibular joint (TMJ) region, limited mouth opening, and joint clicking (Emodi-Perlman et al. 2023).

In line with a biopsychosocial framework, psychosocial factors may be associated with behavioral and clinical manifestations in children. In this context, anxiety symptoms may be linked to increased masticatory muscle activity, which could contribute to the worsening of pain and dysfunction (Bulanda et al., 2021; Emodi-Perlman et al. 2023; do Nascimento et al. 2024). Therefore, this study aimed to explore the association between anxiety, symptoms of temporomandibular disorders, and awake bruxism in children.

## Materials and methods

This observational cross-sectional study was submitted to the Research Ethics Committee of the Federal University of Alagoas (UFAL) under protocol number 6,668,063. The sample size calculation was performed using Epi Info™ software (Centers for Disease Control and Prevention), considering the prevalence of anxiety in children of 23.3% as a parameter, with a 95% confidence interval and a standard error of 5%. Based on this calculation, the minimum sample size required for this investigation was established at 274 children between 7 and 12 years of age. Participant recruitment took place in public schoolyards in the city of Maceió, Alagoas, northeastern Brazil. Clinical evaluation and data collection took place from November 2023 to January 2024.

Children were eligible to participate if they presented erupted permanent first molars and upper and lower incisors, demonstrated sufficient cognitive ability to understand and answer the study instruments, and provided assent along with informed consent signed by their parents or legal guardians. Children were excluded if they had neurological, cognitive, or psychiatric disorders that could interfere with communication; had craniofacial anomalies, orthodontic appliances, or extensive tooth loss that compromised clinical evaluation; were undergoing psychological or pharmacological treatment for anxiety or depression; had chronic orofacial pain, trauma, or recent dental procedures within the preceding 30 days; or refused participation or lacked parental consent.

To assess sociodemographic data, questions regarding the students’ sex and age were used. The anxiety variable was assessed using the State–Trait Anxiety Inventory for Children. This scale was developed by Spielberger and Edwards (1973) and validated in Brazil by Biaggio et al. (1980). It is widely used to measure anxiety levels in children through its adapted version, STAI-C (Spielberger and Edwards 1973). It consists of two subscales: one that assesses trait anxiety (general tendency toward anxiety) and the other that assesses state anxiety (situational anxiety) (Vos et al. 2020). Each subscale consists of 20 items, totaling 40 items, and each item uses a 3-point Likert scale (never, sometimes, often). For this study, children who achieved a minimum score of 31 points were considered anxious.

Awake bruxism was assessed using subject-based (self-report) and clinically based (clinical examination) measures, according to the International Consensus on the Assessment of Bruxism (Verhoeff et al. 2025).

Temporomandibular disorder (TMD) symptoms were assessed through structured interviews conducted with the children, following a simplified clinical approach suitable for epidemiological studies. The evaluation included the observation of jaw movements, measurement of maximum mouth opening, and assessment of pain on palpation of the temporal and masticatory muscles. In addition, the presence of headaches attributed to TMD was recorded when pain in the temporal region was influenced by mandibular movement or function. Children who reported these symptoms were classified as presenting TMD symptoms. This methodology was based on previous pediatric studies that investigated musculoskeletal symptoms of the masticatory system using simplified clinical criteria for the identification of TMD (AAPD, 2024).

It is important to acknowledge that children may present inherent limitations, as they often lack full mastery of language and may have difficulty recognizing and interpreting their own signs and symptoms. To minimize interviewer bias and ensure comprehension, interviews were conducted using simplified and age-appropriate language, with the presence of parents or guardians when necessary. The questions were read aloud slowly, and everyday examples were provided to facilitate understanding. An intraoral clinical examination was performed to identify enamel wear, visible dentin exposure and/or loss of crown height, and the presence of linea alba on the buccal mucosa or lateral surface of the tongue (Verhoeff et al. 2025).

After data collection, variables were categorized and statistically analyzed using Jamovi software and SPSS (Statistical Package for Social Sciences) version 23. The Kolmogorov–Smirnov test was used to verify the normal distribution of the data. The Chi-square test was used to test the association between two categorical variables, or Fisher’s exact test when conditions were required.

Additionally, a binary logistic regression model (enter method) was applied to identify independent predictors of anxiety (dependent variable), including the variables awake bruxism, occlusal wear (OW), TMD symptoms, gender, and age. The model fit was assessed using the deviance, the Akaike Information criterion (AIC), and the Cox and Snell *R*^2^. Results were expressed as odds ratios (OR) with 95% confidence intervals. The significance level was set at p < 0.05. Finally, the assumption of multicollinearity was verified using the variance inflation factor (VIF) and tolerance.

## Results

Two hundred and seventy-four children of both genders were analyzed. Students’ age ranged from 7 to 12 years, with a mean of 9.22 ± 1.41 years. The sample was equally distributed between the sexes. Table 1 highlights that 23.3% of the sample presented anxiety, 4.3% had TMD symptoms, and 18.2% presented awake bruxism.

**Table 1:** Occlusal wear assessment

Variable	*n* (%)
Total	274 (100,0)
Anxiety	
Present	64 (23,3)
Absent	210 (76,7)
TMD symptoms	
Present	12 (4,3)
Absent	262 (95,7)
Bruxism	
Present	50 (18,2)
Absent	224 (81,8)

Table 2 describes that anxiety was associated with sex (*p* = 0.005), TMD symptoms (*p* = 0.008), and tooth wear (*p* = 0.005).

**Table 2:** Assessment of anxiety according to the variables sex, bruxism, and TMD

Variable	Anxiety				
	No	Yes	Total	*p*-value	OR (95% CI)
Sex					
Feminine	91	46	137	P(1) = 0.005*	2.55 (1.56 to 4.17)
Masculine	119	18	137		1
Age					
7 to 9	117	36	153	P(1) = 0.970	1
10 to 12	92	28	120		0.98 (0.56 to 1.73)
TMD symptoms					
No	205	57	262	P(2) = 0.008*	1
Yes	5	7	12		5.05 (1.54 to 16.46)
Awake bruxism					
No	177	47	224	P(1) = 0.049	1
Yes	33	17	50		1.94 (0.99 to 3.78)

Chi-square test

Fisher’s exact test

(*) Significant association at 0.05%

### Binary logistic regression

To identify independent predictors of anxiety, a binary logistic regression model was applied. The model presented deviance = 260, AIC = 272, and a Cox and Snell *R*^2^ = 0.123, suggesting that approximately 12.3% of the variance in anxiety is explained by the included predictors. Overall, this indicates a limited predictive capacity of the model for anxiety. The model results (Table 3) showed that, after adjusting for the remaining variables, the independent and significant predictors of anxiety were occlusal wear (OW), TMD symptoms, and gender. Occlusal wear was a highly significant predictor (*p* = 0.003), conferring 6.92 times higher odds of anxiety (OR = 6.92) in children who presented the condition. TMD symptoms were significant predictors (*p* = 0.036), with affected children presenting 4.38 times higher odds of having anxiety (OR = 4.38). Sex was a strongly significant predictor (*p* < 0.001), with male sex presenting 4.76 times higher odds of anxiety (OR = 4.76) compared to the female sex (reference group). In contrast, awake bruxism (*p* = 0.483) and age (*p* = 0.124) were not independent predictors of anxiety in the studied model. In the multicollinearity analysis, there was no evidence of multicollinearity among the independent variables, with all VIF values below 5 and tolerance above 0.7, confirming the stability of the regression model (Tables 4 and 5).

**Table 3:** Model fit indices for the binary logistic regression

Measure	Value
Deviance	260
AIC	272
*R*^2^ Cox and Snell	0.123

Model fit indices for the logistic regression predicting anxiety. The model explains approximately 12.3% of the variance in the dependent variable

**Table 4:** Logistic regression coefficients for anxiety

		Confidence interval to 95%				
Predictor	Coefficient	Lower limit	Upper limit	Standard error	Z	p
	(β)					
Intercept (α)	-3.969	-61.588	-1.779	1.117	-3.553	< .001
Bruxism	0.308	-0.5535	1.170	0.440	0.701	0.483
OW	1.935	0.6494	3.220	0.656	2.950	0.003
TMD	1.478	0.0985	2.858	0.704	2.100	0.036
Gender	1.560	0.8703	2.249	0.352	4.434	< .001
Age	0.171	-0.0467	0.388	0.111	1.539	0.124

Binary logistic regression coefficients for predicting anxiety. Estimates represent the log odds of “Anxiety = 1.” Odds ratios indicate how much each predictor increases the likelihood of anxiety

**Table 5:** Collinearity statistics of independent variables

	VIF	Tolerance
Bruxism	1.35	0.741
OW	1.21	0.828
TMD	1.18	0.846
Gender	1.13	0.885
Age	1.03	0.967

Variance inflation factor (VIF) values indicate no concerning multicollinearity among predictors

### Summary of findings

Awake bruxism and age were not independently associated with anxiety in the adjusted model. Overall, tooth wear, TMD symptoms, and male sex were independently associated with anxiety in children aged 7–12 years. These findings should be interpreted as associations, and the cross-sectional design and the limited variance explained by the model should be considered when interpreting these results.

## Discussion

Anxiety disorders are the most prevalent mental health conditions worldwide and pose a substantial global health burden (Rapee et al. 2023; Salari et al. 2024). The present study found a slightly higher prevalence of anxiety compared to other works in the field. A recent systematic review identified a 4.7% prevalence of childhood anxiety, while other studies reported rates ranging from 5 to 10% in the 6- to 18-year-old population (Rapee et al. 2023). As seen, prevalence varies widely across studies, which may be explained by the influence of socioeconomic factors (Jefferies and Ungar 2020). Additionally, family history, place of residence, and medical conditions contribute to the development of anxiety disorders (Rapee et al. 2023).

In contrast to the findings of the present study, studies show that females are more susceptible to anxiety disorders than males, as demonstrated by Yoon et al. (2023), who found that in children aged 11–14 years, overall levels of mental health problems were higher in girls than in boys, and that girls tend to show clearer signs of mental distress with advancing age. This gender difference may be partially explained by the earlier cognitive and emotional development observed in girls. Psychological distress often results from physical, social, emotional, and hormonal changes, especially at the onset of puberty, which may justify the increasing disparity. Moreover, females are more likely to internalize stress, contributing to a greater vulnerability to anxiety disorders (Jefferies and Ungar 2020; Yoon et al. 2023).

Conversely, Schmidt and Hansson (2024) highlighted that boys may experience substantial stress throughout development; however, sociocultural norms and gender-related stigma can hinder emotional recognition and expression. As a result, boys often rely on distraction-oriented coping strategies, such as engagement in physical activities, and may require supportive environments to develop emotional awareness and expression. These factors may contribute to the underreporting of psychological distress, particularly in younger populations. Additionally, methodological aspects, including cultural influences, potential sampling bias, and the characteristics of the assessment instrument, may also help explain the observed discrepancy. Anxiety symptoms also demonstrate a propensity for oral parafunctional behaviors such as teeth clenching and grinding (Colonna et al. 2021).

In our adjusted model, awake bruxism did not emerge as an independent predictor of anxiety. This contrasts with previous reports describing associations between anxiety-related symptoms and daytime clenching/mandible bracing (Colonna et al. 2021; Saracutu et al. 2024). Differences in populations and assessment approaches may contribute to variability across studies (Verhoeff et al. 2025). Our sample involved children aged 7–12 years with less cumulative exposure to emotional stressors over time, which may attenuate the association between AB and anxiety. Manfredini et al. (2016) emphasize that bruxism commonly reflects psychological dimensions.

Unfortunately, the assessment of bruxism in pediatric populations remains a methodological challenge, mainly because it relies heavily on parental reports, which are often unreliable, as noted by Huynh et al. (2015) who found substantial underreporting of bruxism by parents, suggesting limited awareness and understanding of the condition. This limitation must be considered when interpreting studies that rely on self-report instruments. STAB (Standardized Tool for the Assessment of Bruxism) (Manfredini et al. 2022) considers instrumental assessment such as polysomnography and electromyography as the gold standard for assessing bruxism (previously named definite bruxism), but these examinations are not widely accessible due to their high cost. STAB (2022) also emphasizes the importance of considering the multifactorial nature of bruxism when determining whether it can be classified as a deleterious habit, a protective factor, or merely neutral behavior.

The study by Saracutu, Bracci, Colonna et al. (2024) explored the association between oral behaviors and psychological symptoms using the PHQ-4, an ultra-brief and validated screening instrument for anxiety and depression that can also be applied to children and adolescents. In their sample, participants reported mild to severe emotional distress, showing moderate to strong correlations particularly with mandible bracing and teeth clenching. In children, these symptoms may represent a manifestation of emotional vulnerability and increase of muscle tension, and thus constitute a potential risk factor for TMD development in adulthood. Considering the multifactorial etiology of TMDs, anxiety may contribute to the development or worsening of TMD symptoms (Mélou et al. 2023; Thomas et al. 2023; Nazzal et al. 2023).

In the present study, TMD symptoms showed a low prevalence in our sample. Prevalence in this age group remains poorly established in the literature, with estimates varying widely from 4 to 68% (Rongo et al. 2021). This large variation likely stems from the fact that TMD is not commonly diagnosed in childhood, as children do not always report or seek care for TMD complaints (Thomas et al. 2023). Despite the low prevalence in our sample, children with TMD symptoms were five times more likely to present with anxiety symptoms. Similarly, Nazzal et al. (2023) found that children with social phobia, a type of anxiety disorder, were more likely to develop TMD, reinforcing the association between anxiety and TMD.

Further studies are needed to evaluate anxiety as a risk factor for the onset and persistence of TMD in children and into adulthood, across its various subtypes (Salari et al. 2024). Currently, the IADR principles and recommendations and the Diagnostic Criteria for Temporomandibular Disorders (DC/TMD) are considered the gold standard for TMD assessment. Their validation for pediatric populations is still under development (Rongo et al. 2021).

Considering the studied variables, awake bruxism was not independently associated with anxiety in our adjusted model, although it is often discussed in the literature as part of a broader biopsychosocial context involving anxiety-related symptoms and TMD (Nazzal et al. 2023). Proposed explanations suggest that emotional stress and anxiety may be accompanied by increased masticatory muscle tension and oral parafunctional behaviors, which could be relevant to the presence of painful symptoms in susceptible individuals (Yazıcıoğlu and Çam Ray, 2022; Nazzal et al. 2023). However, these relationships should be interpreted cautiously, particularly given differences in assessment approaches and the cross-sectional nature of the available evidence, and longitudinal studies are needed to clarify temporal pathways between anxiety symptoms, oral behaviors, and TMD symptoms in children (Rongo et al. 2021).

This study has limitations inherent to its cross-sectional design, which preclude establishing cause-and-effect relationships among the studied variables. It is worth noting that children’s oral health assessments are influenced by socioeconomic and psychological conditions. Furthermore, non-age-specific questionnaires were used in the evaluation of TMD symptoms, which may underestimate or overestimate the prevalence.

## Conclusion

Based on this cross-sectional study, anxiety affected almost a quarter of the study sample and was associated with sex, TMD symptoms, and tooth wear, suggesting that psychosocial and behavioral factors may be relevant to the presentation of orofacial conditions in this population. Awake bruxism was not identified as an independent predictor of anxiety. Overall, these findings support an association between anxiety and specific orofacial clinical features. However, given the exploratory and associative nature of the study and the limited variance explained by the model, the results should be interpreted with caution and do not allow causal inferences.

## Data Availability

No datasets were generated or analyzed during the current study.
